# [Bis(pyridin-2-ylmeth­yl) ether]trichlorido­rhodium(III) dichloro­methane monosolvate: unusual hydrolysis of the methyl­ene bridge in (pyrazolylmeth­yl)pyridine

**DOI:** 10.1107/S1600536811027644

**Published:** 2011-07-16

**Authors:** Stephen O. Ojwach, Bernard Omondi, James Darkwa

**Affiliations:** a Department of Chemistry, University of Johannesburg, Auckland Park Kingsway Campus, PO Box 524, Johannesburg, South Africa; b School of Chemistry, University of KwaZulu-Natal, Westville Campus, Private Bag X54001, Durban 4000, South Africa

## Abstract

In the title compound, [RhCl_3_(C_12_H_12_N_2_O)]·CH_2_Cl_2_, the Rh^III^ atom shows a slightly distorted octa­hedral geometry being coordinated by two N atoms and one O atom from the 2,2′-(oxydimethanedi­yl)dipyridine ligand and three Cl atoms. Two Cl atoms adopt a *trans* arrangement to the two pyridyl N atoms, while the third Cl atom and the O atoms occupy the axial site. The Rh—Cl bonds that are *trans* to the pyridyl N atoms are slightly longer than the Rh—Cl bond distance *trans* to the O atom.

## Related literature

For hydrogenation of olefins, see: Samec *et al.* (2006 ▶); Xu *et al.* (2009 ▶); Chalid *et al.* (2011 ▶); Liu *et al.* (2011 ▶). For multidentate N-containing ligands, see: Dayan & Centikaya (2007 ▶); Deng *et al.* (2005 ▶). For pyrazolyl-based transition metal complexes as catalysts, see: Ojwach & Darkwa (2010 ▶) and references therein. For structures bearing the 2,2′-(oxydimethanedi­yl)dipyridine ligand, see: Nanty *et al.* (2000 ▶) and references therein. 
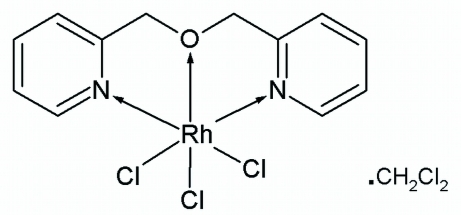


## Experimental

### 

#### Crystal data

[RhCl_3_(C_12_H_12_N_2_O)]·CH_2_Cl_2_
*M* *_r_* = 494.42
Monoclinic, 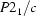

*a* = 9.5360 (18) Å
*b* = 12.527 (2) Å
*c* = 14.340 (3) Å
β = 95.071 (4)°
*V* = 1706.3 (6) Å^3^
*Z* = 4
Mo *K*α radiation
μ = 1.78 mm^−1^
*T* = 100 K
0.14 × 0.13 × 0.05 mm

#### Data collection

Bruker X8 APEXII 4K KappaCCD diffractometer
Absorption correction: multi-scan (*SADABS*; Bruker, 2007 ▶) *T*_min_ = 0.788, *T*_max_ = 0.916
30694 measured reflections
4276 independent reflections
2925 reflections with *I* > 2σ(*I*)
*R*_int_ = 0.102

#### Refinement

*R*[*F* ^2^ > 2σ(*F* ^2^)] = 0.048
*wR*(*F* ^2^) = 0.139
*S* = 1.08
4276 reflections
199 parameters
H-atom parameters constrained
Δρ_max_ = 1.50 e Å^−3^
Δρ_min_ = −1.25 e Å^−3^

### 

Data collection: *APEX2* (Bruker, 2007 ▶); cell refinement: *SAINT-Plus* (Bruker, 2007 ▶); data reduction: *SAINT-Plus* and *XPREP* (Bruker, 2007 ▶); program(s) used to solve structure: *SHELXS97* (Sheldrick, 2008 ▶); program(s) used to refine structure: *SHELXL97* (Sheldrick, 2008 ▶); molecular graphics: *DIAMOND* (Brandenburg & Putz, 2005 ▶) and *ORTEP-3* (Farrugia, 1997 ▶); software used to prepare material for publication: *WinGX* (Farrugia, 1999 ▶).

## Supplementary Material

Crystal structure: contains datablock(s) global, I. DOI: 10.1107/S1600536811027644/om2440sup1.cif

Structure factors: contains datablock(s) I. DOI: 10.1107/S1600536811027644/om2440Isup2.hkl

Additional supplementary materials:  crystallographic information; 3D view; checkCIF report

## Figures and Tables

**Table 1:** Selected bond lengths (Å)

N1—Rh1	2.037 (5)
N2—Rh1	2.031 (5)
O1—Rh1	2.069 (4)
Cl1—Rh1	2.3479 (15)
Cl2—Rh1	2.2941 (15)
Cl3—Rh1	2.3315 (15)

**Fig. 1.**
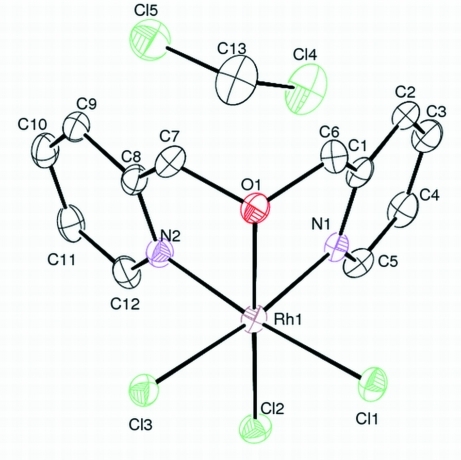
The asymmetric unit of the title compound with displacement ellipsoids drawn at the 50% probability level. Hydrogen atoms are omitted for clarity.

## supplementary crystallographic information

### Comment

Transition metal catalyzed hydrogenation of olefins constitutes one of the most important reactions in fine chemicals and petroleum industries (Samec *et al.*, 2006, Xu *et al.*, 2009). To date most hydrogenation catalysts reported in literature are based on phosphine ruthenium and rhodium complexes (Chalid *et al.*, 2011, Liu *et al.*, 2011). Recently multidentate nitrogen-containing ligands such as 2,6-bis(imino)pyridine (Dayan & Centikaya, 2007, and 2,6(3,5-dimethylpyrazol-1-yl)pyridine (Deng *et al.*, 2005), have attracted much attention, and have been used as effective catalysts for transfer hydrogenation of ketones. As part of our investigation of pyrazolyl-based transition metal complexes as catalysts for various olefin transformations (Ojwach and Darkwa, 2010 and references therein), we are currently exploring the ability of (pyrazolylmethyl)pyridine ruthenium and rhodium complexes to catalyze olefin hydrogenation reactions. On one such attempt to synthesize the rhodium complex of 2-(3,5-dimethylpyrazol-1-ylmethyl)pyridine, the title compound was obtained. This transformation points to the possible hydrolysis of the 2-(3,5-dimethylpyrazol-1-ylmethyl)pyridine compound promoted by RhCl_3_.6H_2_O salt to form the (pyridinylmethyl)ether ligand in the title compound.

In the title compound [RhCl_3_(C_12_H_12_N_2_O)].CH_2_Cl_2_ the asymmetric unit contains one molecule of the Rh^III^ complex and a dichloromethane molecule of solvation (Fig. 1). In the structure, the Rh atom center is in an octahedral environment with two nitrogen atoms from 2,2'-(oxydimethanediyl)dipyridine ligand *trans* to two chlorine atoms in the equatorial position. The axial positions are occupied by a third chlorine atom and the oxygen atom from 2,2'-(oxydimethanediyl)dipyridine. 2,2'-(oxydimethanediyl)dipyridine acts as an *N,O,N'* tridentate ligand in which the angles around the the Rh metal center are close to orthogonal (see Table 1). The bond distances for the Cl atoms to the Rh atom are longer for the Cl atoms *trans* to the pyridyl N atoms of the 2,2'-(oxydimethanediyl)dipyridine ligand (2.3315 (15) and 2.3479 (15) Å) as compared to the distance of the Cl atom *trans* to the O atom (2.2941 (5) Å). A similar trend is observed in closely related compounds with the same ligand system where metal–ligand bond distance that is *trans* to the ether ligand was found to be statistically shorter than the distance between the *trans* ligands to pyridyl N atoms (the *trans* effect) (Nanty *et al.*, 2000 and references therein). The Rh atom is slightly off the equatorial plane N_2_Cl_2_ and is inclined toward the axial Cl atom as the bond angles N–Rh–Cl would suggest (172.82 (13) and 172.89 (14)°). The O–Rh–Cl bond angle is 176.29 (12)° also pointing to a slight inclination of the Rh toward the equatorial Cl atoms.

### Experimental

To a solution of RhCl_3_.6H_2_O (0.20 g, 0.60 mmol) in MeOH (10 ml) was added a solution of 2-(3,5-dimethylpyazol-1-ylmethyl)pyridine (0.12 g, 0.60 mmol) in MeOH (10 ml) and the resultant orange solution was stirred for 24 h. After the reaction period, the solution was filtered off and solvent removed *in vacuo* to afford an orange solid. Recrystallization of the crude product from chloroform gave single crystals suitable for X-ray analysis. The crystals were insoluble in most organic solvents. Yield = 0.09 g (40%).

### Refinement

H atoms on C atoms were placed in idealized positions (C–H = 0.95) for aromatic H atoms and (C–H = 0,99) for methylenic H atoms and refined as riding atoms with *U*_iso_(H) = 1.5*U*_eq_(C).

### Crystal data

**Table:**

[RhCl_3_(C_12_H_12_N_2_O)]·CH_2_Cl_2_	*F*(000) = 976
*M**_r_* = 494.42	*D*_x_ = 1.925 Mg m^−^^3^
Monoclinic, *P*2_1_/*c*	Mo *K*α radiation, λ = 0.71073 Å
Hall symbol: -P 2ybc	Cell parameters from 31705 reflections
*a* = 9.5360 (18) Å	θ = 2.1–28.6°
*b* = 12.527 (2) Å	µ = 1.78 mm^−^^1^
*c* = 14.340 (3) Å	*T* = 100 K
β = 95.071 (4)°	Block, brown
*V* = 1706.3 (6) Å^3^	0.14 × 0.13 × 0.05 mm
*Z* = 4	

### Data collection

**Table:**

Bruker X8 APEXII 4K KappaCCD diffractometer	2925 reflections with *I* > 2σ(*I*)
graphite	*R*_int_ = 0.102
φ and ω scans	θ_max_ = 28.6°, θ_min_ = 2.1°
Absorption correction: multi-scan (*SADABS*; Bruker, 2007)	*h* = −12→12
*T*_min_ = 0.788, *T*_max_ = 0.916	*k* = −15→16
30694 measured reflections	*l* = −19→19
4276 independent reflections	

### Refinement

**Table:**

Refinement on *F*^2^	Primary atom site location: structure-invariant direct methods
Least-squares matrix: full	Secondary atom site location: difference Fourier map
*R*[*F*^2^ > 2σ(*F*^2^)] = 0.048	Hydrogen site location: inferred from neighbouring sites
*wR*(*F*^2^) = 0.139	H-atom parameters constrained
*S* = 1.08	*w* = 1/[σ^2^(*F*_o_^2^) + (0.0602*P*)^2^ + 6.0697*P*] where *P* = (*F*_o_^2^ + 2*F*_c_^2^)/3
4276 reflections	(Δ/σ)_max_ < 0.001
199 parameters	Δρ_max_ = 1.50 e Å^−^^3^
0 restraints	Δρ_min_ = −1.25 e Å^−^^3^

### Special details

**Table:**

Experimental. The intensity data was collected on a Bruker X8 Apex 4 K CCD diffractometer using an exposure time of 20 sec/per frame. A total of 3527 frames were collected with a frame width of 0.5° covering upto θ = 28.57° with 99.8% completeness accomplished.
Geometry. All e.s.d.'s (except the e.s.d. in the dihedral angle between two l.s. planes) are estimated using the full covariance matrix. The cell e.s.d.'s are taken into account individually in the estimation of e.s.d.'s in distances, angles and torsion angles; correlations between e.s.d.'s in cell parameters are only used when they are defined by crystal symmetry. An approximate (isotropic) treatment of cell e.s.d.'s is used for estimating e.s.d.'s involving l.s. planes.
Refinement. Refinement of *F*^2^ against ALL reflections. The weighted *R*-factor *wR* and goodness of fit *S* are based on *F*^2^, conventional *R*-factors *R* are based on *F*, with *F* set to zero for negative *F*^2^. The threshold expression of *F*^2^ > σ(*F*^2^) is used only for calculating *R*-factors(gt) *etc*. and is not relevant to the choice of reflections for refinement. *R*-factors based on *F*^2^ are statistically about twice as large as those based on *F*, and *R*- factors based on ALL data will be even larger. >>> The Following ALERTS were generated <<< Format: alert-number_ALERT_alert-type_alert-level text 973_ALERT_2_B Large Calcd. Positive Residual Density on Rh1 1.53 e A-3 Difference map does not reveal any missing peaks, *R* factor is 4.8% 342_ALERT_3_C Low Bond Precision on C—C Bonds (*x* 1000) Ang.. 9 083_ALERT_2_G *SHELXL* Second Parameter in WGHT Unusually Large. 6.07 960_ALERT_3_G Number of Intensities with *I*. LT. - 2*sig(*I*).. 7 912_ALERT_4_C Missing # of FCF Reflections Above STh/*L*= 0.600 76Noted.

### Fractional atomic coordinates and isotropic or equivalent isotropic displacement parameters (Å^2^)

**Table:**

	*x*	*y*	*z*	*U*_iso_*/*U*_eq_	
C1	0.9453 (6)	0.3170 (5)	0.5707 (4)	0.0220 (12)	
C2	0.9516 (6)	0.3990 (5)	0.6344 (4)	0.0250 (13)	
H2	0.9982	0.3893	0.6952	0.03*	
C3	0.8896 (7)	0.4959 (5)	0.6095 (4)	0.0286 (14)	
H3	0.8936	0.5538	0.6525	0.034*	
C4	0.8218 (7)	0.5068 (5)	0.5210 (4)	0.0284 (14)	
H4	0.7789	0.5727	0.5022	0.034*	
C5	0.8166 (6)	0.4224 (5)	0.4604 (4)	0.0238 (13)	
H5	0.7684	0.43	0.3999	0.029*	
C6	1.0152 (6)	0.2118 (5)	0.5914 (4)	0.0213 (12)	
H6A	1.1158	0.2167	0.5797	0.026*	
H6B	1.0101	0.194	0.6583	0.026*	
C7	0.8409 (6)	0.0651 (5)	0.5762 (4)	0.0240 (13)	
H7A	0.8595	0.0672	0.6452	0.029*	
H7B	0.8459	−0.0103	0.556	0.029*	
C8	0.6967 (6)	0.1079 (5)	0.5489 (4)	0.0217 (12)	
C9	0.5837 (6)	0.0877 (5)	0.6005 (4)	0.0248 (13)	
H9	0.5973	0.05	0.6581	0.03*	
C10	0.4508 (7)	0.1230 (5)	0.5677 (4)	0.0285 (14)	
H10	0.372	0.11	0.6023	0.034*	
C11	0.4352 (6)	0.1776 (5)	0.4833 (4)	0.0284 (14)	
H11	0.3447	0.2014	0.4589	0.034*	
C12	0.5499 (6)	0.1970 (5)	0.4353 (4)	0.0272 (13)	
H12	0.5381	0.2353	0.378	0.033*	
C13	0.5353 (9)	0.4120 (7)	0.6578 (7)	0.053 (2)	
H13A	0.6165	0.3889	0.7009	0.064*	
H13B	0.5467	0.3812	0.5953	0.064*	
N1	0.8778 (5)	0.3292 (4)	0.4846 (3)	0.0179 (10)	
N2	0.6797 (5)	0.1632 (4)	0.4675 (3)	0.0212 (10)	
O1	0.9483 (4)	0.1273 (3)	0.5336 (3)	0.0204 (8)	
Cl1	1.08727 (15)	0.21896 (12)	0.35869 (10)	0.0231 (3)	
Cl2	0.76119 (16)	0.27205 (13)	0.27541 (10)	0.0255 (3)	
Cl3	0.83695 (15)	0.02382 (12)	0.33886 (9)	0.0231 (3)	
Cl4	0.5347 (2)	0.55102 (18)	0.64998 (18)	0.0583 (6)	
Cl5	0.3773 (2)	0.36285 (18)	0.69904 (14)	0.0513 (5)	
Rh1	0.86208 (5)	0.19166 (4)	0.40861 (3)	0.01818 (13)	

### Atomic displacement parameters (Å^2^)

**Table:**

	*U*^11^	*U*^22^	*U*^33^	*U*^12^	*U*^13^	*U*^23^
C1	0.020 (3)	0.023 (3)	0.025 (3)	−0.002 (2)	0.010 (2)	0.002 (2)
C2	0.026 (3)	0.028 (4)	0.021 (3)	−0.007 (3)	0.006 (2)	0.003 (2)
C3	0.037 (4)	0.018 (3)	0.032 (3)	−0.002 (3)	0.014 (3)	−0.004 (2)
C4	0.030 (3)	0.018 (3)	0.039 (3)	0.003 (2)	0.011 (3)	0.002 (3)
C5	0.023 (3)	0.025 (3)	0.024 (3)	0.002 (2)	0.006 (2)	0.006 (2)
C6	0.018 (3)	0.019 (3)	0.026 (3)	−0.001 (2)	0.001 (2)	−0.001 (2)
C7	0.025 (3)	0.023 (3)	0.024 (3)	−0.002 (2)	0.007 (2)	0.005 (2)
C8	0.023 (3)	0.018 (3)	0.024 (3)	−0.003 (2)	0.005 (2)	0.000 (2)
C9	0.026 (3)	0.025 (3)	0.024 (3)	−0.007 (3)	0.007 (2)	−0.002 (2)
C10	0.023 (3)	0.029 (4)	0.035 (3)	−0.006 (3)	0.010 (3)	−0.006 (3)
C11	0.016 (3)	0.029 (4)	0.039 (3)	−0.002 (2)	−0.001 (2)	−0.004 (3)
C12	0.024 (3)	0.027 (4)	0.029 (3)	−0.004 (3)	0.000 (2)	−0.005 (3)
C13	0.042 (5)	0.040 (5)	0.077 (6)	0.013 (4)	0.008 (4)	0.013 (4)
N1	0.016 (2)	0.016 (3)	0.023 (2)	−0.0009 (18)	0.0045 (18)	0.0013 (18)
N2	0.020 (2)	0.021 (3)	0.023 (2)	−0.0013 (19)	0.0039 (19)	−0.0011 (19)
O1	0.018 (2)	0.022 (2)	0.0219 (19)	−0.0005 (16)	0.0038 (15)	0.0005 (16)
Cl1	0.0195 (7)	0.0223 (8)	0.0283 (7)	−0.0014 (5)	0.0060 (5)	−0.0002 (5)
Cl2	0.0257 (7)	0.0280 (8)	0.0227 (7)	0.0011 (6)	0.0018 (5)	0.0037 (6)
Cl3	0.0247 (7)	0.0205 (8)	0.0244 (6)	−0.0026 (6)	0.0041 (5)	−0.0008 (5)
Cl4	0.0419 (12)	0.0495 (13)	0.0855 (16)	0.0110 (9)	0.0173 (11)	0.0111 (11)
Cl5	0.0472 (12)	0.0535 (13)	0.0525 (11)	0.0010 (9)	0.0010 (9)	0.0028 (9)
Rh1	0.0174 (2)	0.0170 (2)	0.0204 (2)	−0.00075 (18)	0.00326 (15)	0.00144 (18)

### Geometric parameters (Å, °)

**Table:**

C1—N1	1.349 (7)	C8—C9	1.383 (8)
C1—C2	1.373 (8)	C9—C10	1.385 (9)
C1—C6	1.495 (8)	C9—H9	0.95
C2—C3	1.384 (9)	C10—C11	1.387 (9)
C2—H2	0.95	C10—H10	0.95
C3—C4	1.380 (9)	C11—C12	1.365 (8)
C3—H3	0.95	C11—H11	0.95
C4—C5	1.367 (9)	C12—N2	1.350 (8)
C4—H4	0.95	C12—H12	0.95
C5—N1	1.336 (7)	C13—Cl4	1.745 (9)
C5—H5	0.95	C13—Cl5	1.777 (9)
C6—O1	1.456 (7)	C13—H13A	0.99
C6—H6A	0.99	C13—H13B	0.99
C6—H6B	0.99	N1—Rh1	2.037 (5)
C7—O1	1.462 (7)	N2—Rh1	2.031 (5)
C7—C8	1.496 (8)	O1—Rh1	2.069 (4)
C7—H7A	0.99	Cl1—Rh1	2.3479 (15)
C7—H7B	0.99	Cl2—Rh1	2.2941 (15)
C8—N2	1.355 (7)	Cl3—Rh1	2.3315 (15)
			
N1—C1—C2	120.8 (5)	C12—C11—C10	120.0 (6)
N1—C1—C6	116.8 (5)	C12—C11—H11	120
C2—C1—C6	122.3 (5)	C10—C11—H11	120
C1—C2—C3	119.5 (6)	N2—C12—C11	121.5 (6)
C1—C2—H2	120.2	N2—C12—H12	119.2
C3—C2—H2	120.2	C11—C12—H12	119.2
C4—C3—C2	118.6 (6)	Cl4—C13—Cl5	111.6 (4)
C4—C3—H3	120.7	Cl4—C13—H13A	109.3
C2—C3—H3	120.7	Cl5—C13—H13A	109.3
C5—C4—C3	119.8 (6)	Cl4—C13—H13B	109.3
C5—C4—H4	120.1	Cl5—C13—H13B	109.3
C3—C4—H4	120.1	H13A—C13—H13B	108
N1—C5—C4	121.3 (6)	C5—N1—C1	119.9 (5)
N1—C5—H5	119.3	C5—N1—Rh1	126.1 (4)
C4—C5—H5	119.3	C1—N1—Rh1	113.5 (4)
O1—C6—C1	111.2 (5)	C12—N2—C8	119.4 (5)
O1—C6—H6A	109.4	C12—N2—Rh1	126.6 (4)
C1—C6—H6A	109.4	C8—N2—Rh1	114.0 (4)
O1—C6—H6B	109.4	C6—O1—C7	116.0 (4)
C1—C6—H6B	109.4	C6—O1—Rh1	109.3 (3)
H6A—C6—H6B	108	C7—O1—Rh1	109.3 (3)
O1—C7—C8	111.2 (5)	N2—Rh1—N1	87.23 (19)
O1—C7—H7A	109.4	N2—Rh1—O1	81.99 (17)
C8—C7—H7A	109.4	N1—Rh1—O1	82.03 (17)
O1—C7—H7B	109.4	N2—Rh1—Cl2	96.40 (14)
C8—C7—H7B	109.4	N1—Rh1—Cl2	94.57 (13)
H7A—C7—H7B	108	N2—Rh1—Cl3	87.75 (14)
N2—C8—C9	121.0 (6)	O1—Rh1—Cl3	92.19 (12)
N2—C8—C7	116.5 (5)	Cl2—Rh1—Cl3	91.09 (6)
C9—C8—C7	122.4 (5)	N1—Rh1—Cl1	90.78 (13)
C8—C9—C10	119.5 (6)	O1—Rh1—Cl1	90.98 (11)
C8—C9—H9	120.2	Cl2—Rh1—Cl1	90.56 (5)
C10—C9—H9	120.2	Cl3—Rh1—Cl1	93.59 (5)
C9—C10—C11	118.5 (6)	N1—Rh1—Cl3	172.82 (13)
C9—C10—H10	120.7	N2—Rh1—Cl1	172.89 (14)
C11—C10—H10	120.7	O1—Rh1—Cl2	176.29 (12)
			
N1—C1—C2—C3	−0.9 (9)	C12—N2—Rh1—N1	87.7 (5)
C6—C1—C2—C3	177.4 (5)	C8—N2—Rh1—N1	−89.7 (4)
C1—C2—C3—C4	0.6 (9)	C12—N2—Rh1—O1	170.1 (5)
C2—C3—C4—C5	0.4 (9)	C8—N2—Rh1—O1	−7.3 (4)
C3—C4—C5—N1	−1.0 (9)	C12—N2—Rh1—Cl2	−6.6 (5)
N1—C1—C6—O1	−25.6 (7)	C8—N2—Rh1—Cl2	176.1 (4)
C2—C1—C6—O1	156.1 (5)	C12—N2—Rh1—Cl3	−97.4 (5)
O1—C7—C8—N2	24.6 (7)	C8—N2—Rh1—Cl3	85.2 (4)
O1—C7—C8—C9	−158.8 (5)	C12—N2—Rh1—Cl1	161.6 (9)
N2—C8—C9—C10	1.2 (9)	C8—N2—Rh1—Cl1	−15.8 (15)
C7—C8—C9—C10	−175.2 (6)	C5—N1—Rh1—N2	−84.2 (5)
C8—C9—C10—C11	0.1 (9)	C1—N1—Rh1—N2	88.5 (4)
C9—C10—C11—C12	−1.1 (9)	C5—N1—Rh1—O1	−166.5 (5)
C10—C11—C12—N2	0.8 (10)	C1—N1—Rh1—O1	6.2 (4)
C4—C5—N1—C1	0.7 (8)	C5—N1—Rh1—Cl2	12.0 (5)
C4—C5—N1—Rh1	173.0 (4)	C1—N1—Rh1—Cl2	−175.3 (3)
C2—C1—N1—C5	0.3 (8)	C5—N1—Rh1—Cl3	−129.9 (10)
C6—C1—N1—C5	−178.1 (5)	C1—N1—Rh1—Cl3	42.8 (13)
C2—C1—N1—Rh1	−172.9 (4)	C5—N1—Rh1—Cl1	102.6 (4)
C6—C1—N1—Rh1	8.7 (6)	C1—N1—Rh1—Cl1	−84.7 (4)
C11—C12—N2—C8	0.6 (9)	C6—O1—Rh1—N2	−107.8 (3)
C11—C12—N2—Rh1	−176.7 (5)	C7—O1—Rh1—N2	20.1 (4)
C9—C8—N2—C12	−1.6 (9)	C6—O1—Rh1—N1	−19.5 (3)
C7—C8—N2—C12	175.1 (5)	C7—O1—Rh1—N1	108.4 (4)
C9—C8—N2—Rh1	176.0 (4)	C6—O1—Rh1—Cl2	−43 (2)
C7—C8—N2—Rh1	−7.4 (7)	C7—O1—Rh1—Cl2	84.6 (19)
C1—C6—O1—C7	−95.4 (5)	C6—O1—Rh1—Cl3	164.8 (3)
C1—C6—O1—Rh1	28.7 (5)	C7—O1—Rh1—Cl3	−67.3 (3)
C8—C7—O1—C6	95.3 (5)	C6—O1—Rh1—Cl1	71.2 (3)
C8—C7—O1—Rh1	−28.7 (5)	C7—O1—Rh1—Cl1	−161.0 (3)
